# Therapy-Associated Vitiligo: Hypopigmentation Secondary to Programmed Cell Death Protein 1 Inhibitors, Interferon Alfa-2b, Cytotoxic T-Lymphocyte-Associated Protein 4 Inhibitors, and B-Raf/Mitogen-Activated Protein Kinase Kinase Inhibitors

**DOI:** 10.7759/cureus.112042

**Published:** 2026-07-04

**Authors:** Kritin K Verma, Sreeya Reddy, Caleb Beckham, Kevin T Nguyen, Justin Raman, Matthew Olagbenro, Floyd A Pirtle, Michelle Tarbox, Alba Posligua, Sancy A Leachman, Seemal Desai

**Affiliations:** 1 Dermatology, Texas Tech University Health Sciences Center, Lubbock, USA; 2 Dermatology, Texas Tech University Health Sciences Center El Paso Paul L. Foster School of Medicine, El Paso, USA; 3 Dermatology, University of Alabama, Birmingham, USA; 4 Dermatology, Texas Tech University Health Sciences Center School of Medicine, Lubbock, USA; 5 Dermatology, University of Utah School of Medicine, Salt Lake City, USA; 6 Dermatology, Innovative Dermatology, Plano, USA; 7 Dermatology, The University of Texas Southwestern, Dallas, USA

**Keywords:** braf inhibitors, ctla-4 inhibitor, ifn-alpha, malignant melanoma, mek inhibitor, melanoma treatment, pd-1 inhibitors, treatment-associated vitiligo, vitiligo

## Abstract

Background

Malignant melanoma (MM) arises from melanocytes, and vitiligo is an autoimmune condition targeting these cells. Although an association between MM and vitiligo has been proposed, underlying mechanisms remain unclear. Because several melanoma treatments modulate immune responses, this study evaluated the relationship between MM and vitiligo across commonly used systemic therapies.

Methods

In September 2025, using the TriNetX network, patients with MM receiving programmed cell death protein 1 (PD-1) inhibitors, interferon alpha-2b (IFN-α2b), B-Raf serine/threonine kinases (BRAF)/ mitogen-activated protein kinase kinases (MEK) inhibitors, or cytotoxic T-lymphocyte-associated protein 4 (CTLA-4) inhibitors were identified and 1:1 propensity score-matched to patients without MM receiving the same therapy. Demographics and vitiligo incidence were analyzed using risk ratios (RRs) and 95% confidence intervals (CIs) via Wald’s method. Counts <10 were suppressed per platform guidelines.

Results

Baseline demographics were well balanced between MM and matched non-MM patients. MM was significantly associated with higher vitiligo risk among those treated with PD-1 inhibitors (RR: 24.84; 95% CI: 16.92-36.45), IFN-α2b (RR: 3.10; 95% CI: 1.53-6.30), and CTLA-4 inhibitors (RR: 32.04; 95% CI: 18.05-56.88). In the BRAF/MEK cohort, vitiligo occurred only in patients with MM, preventing RR calculation.

Conclusions

Across multiple therapeutic classes, MM consistently conferred a greater risk of developing vitiligo compared with matched non-MM patients. The strong associations observed with immune-modulating agents support vitiligo as a potential marker of antitumor immune activation rather than a coincidental adverse event. Even rare cases in BRAF/MEK-treated patients suggest that targeted therapy may also influence melanocyte-directed immunity. Further prospective studies are needed to characterize the clinical and immunologic significance of treatment-associated vitiligo.

## Introduction

Malignant melanoma (MM) is an increasingly common and aggressive skin cancer arising from the malignant transformation of pigment-producing cells known as melanocytes [1-2]. Similarly, vitiligo is an autoimmune disease of the skin that targets melanocytes, resulting in patches of depigmented skin [3]. Associations between vitiligo and MM have been postulated in the current literature. However, the pathophysiological etiologies are poorly elucidated [4]. Targeted therapy, such as programmed cell death protein 1 (PD-1) inhibitors, interferon alpha-2b (IFN-α2b), combination B-Raf serine/threonine kinases (BRAF)/mitogen-activated protein kinase kinases (MEK) inhibitors, and cytotoxic T-lymphocyte-associated protein 4 (CTLA-4) inhibitors has been a valuable tool in the treatment of melanoma, along with a multitude of other conditions [5-7]. Numerous well-known side effects have been associated with these medications, such as fatigue, skin reactions, gastrointestinal symptoms, and immune-related endocrine or hepatic dysfunction [8-9]. Using what is known about these medications, we set out to provide an updated analysis and better understanding of the pathogenesis of associations between MM and vitiligo. More specifically, how MM and its treatment may precipitate the onset of vitiligo.

## Materials and methods

Study design

We conducted a retrospective cohort study using de-identified electronic health record data from the TriNetX Research Network to compare the risk of incident vitiligo between patients with melanoma and propensity score-matched patients without melanoma receiving the same systemic therapies. TriNetX is a federated network that aggregates longitudinal clinical data from multiple healthcare organizations, including demographic characteristics, diagnoses, medication exposures, and outcomes.

Inclusion and exclusion criteria

In September 2025, patients with melanoma receiving PD-1 inhibitors, IFN-α2b, combination BRAF/MEK inhibitors, or CTLA-4 inhibitors were identified using International Classification of Diseases (ICD) 10th Clinical Modification (CM) diagnostic codes and treatment records within TriNetX. Melanoma was identified using ICD-10-CM code C43. Incident vitiligo was identified using ICD-10-CM code L80. Patients with an L80 diagnosis or vitiligo documented before therapy initiation were excluded, so that only new-onset vitiligo cases arising after treatment exposure were captured. For each therapeutic category, patients with melanoma were matched 1:1 by propensity score to patients without melanoma receiving the same systemic therapy. A predefined look-back period, minimum treatment exposure, and fixed follow-up window were not specified within the study design. No additional exclusion criteria were applied.

Data collection

Demographic variables were collected for all eligible patients. The primary outcome was incident vitiligo diagnosed after treatment initiation. Only vitiligo cases recorded after the start of therapy were included to minimize inclusion of preexisting disease. Data were identified using standardized coding within the TriNetX platform. Per TriNetX data dissemination guidelines, groups with participant counts fewer than 10 were suppressed. 

Statistical analysis

Propensity score matching was performed in a 1:1 fashion using the variables available for matching within the study, including age, sex, race, and ethnicity, to reduce demographic differences between melanoma and non-melanoma cohorts receiving the same systemic therapies. Risk ratios (RRs) with 95% confidence intervals (CIs) were calculated using Wald’s method. Analyses were performed using TriNetX analytic tools. In accordance with TriNetX guidelines, event counts fewer than 10 were suppressed in the reported tables, although the platform used the underlying data to calculate the reported RR and CI internally.

## Results

Baseline demographic characteristics were well balanced between patients with malignant melanoma (MM) and their matched counterparts without MM, including age, sex, ethnicity, and race (Table 1). The average age at therapy initiation was also comparable across groups.

**Table 1:** Baseline Characteristics in Melanoma and Non-Melanoma Patients Treated with PD-1 Inhibitors, IFN-α2b, CTLA-4 Inhibitors, and BRAF/MEK Inhibitors. MM: malignant melanoma; SD: standard deviation; PD-1i: programmed cell death protein 1 inhibitor; IFN‑α2b: interferon alpha-2b; CTLA-4i: cytotoxic T-lymphocyte-associated protein 4 inhibitor; BRAF/MEKi: B-Raf serine/threonine kinases and mitogen-activated protein kinase kinases inhibitors. **As per TriNetX user guidelines, data with fewer than 10 patients is suppressed from view.

Characteristic	PD-1i		IFN‑α2b		CTLA-4i		BRAF/MEKi	
	MM Cases	MM Controls	MM Cases	MM Controls	MM Cases	MM Controls	MM Cases	MM Controls
Total Patients	17,563 (100%)	17,563 (100%)	1,182 (100%)	1,182 (100%)	7,910 (100%)	7,910 (100%)	2,491 (100.00%)	2,491 (100.00%)
Average Age, SD (Years)	64.7 ± 14.6	64.7 ± 14.7	53.3 ± 16.5	52.8 ± 17.4	63.9 ± 12.8	63.8 ± 12.8	61.8 ± 15.3	59.3 ± 19.5
Female	6,638 (37.80%)	6,638 (37.80%)	467 (39.51%)	453 (38.33%)	2,767 (34.98%)	2,834 (35.83%)	1,172 (47.05%)	1,223 (49.10%)
Male	10,925 (62.20%)	10,925 (62.20%)	715 (60.49%)	729 (61.68%)	5,143 (65.02%)	5,076 (64.17%)	1,319 (52.95%)	1,268 (50.90%)
Not Hispanic or Latino	14,315 (81.51%)	14,327 (81.58%)	919 (77.75%)	943 (79.78%)	6,609 (83.55%)	6,549 (82.79%)	1,934 (77.64%)	1,950 (78.28%)
Hispanic or Latino	483 (2.75%)	486 (2.77%)	25 (2.12%)	21 (1.78%)	286 (3.62%)	300 (3.79%)	125 (5.02%)	133 (5.34%)
White	16,181 (92.13%)	16,184 (92.15%)	1,108 (93.74%)	1,106 (93.57%)	7,209 (91.14%)	7,186 (90.85%)	2,137 (85.79%)	2,137 (85.79%)
Black or African American	236 (1.34%)	238 (1.36%)	13 (1.10%)	13 (1.10%)	124 (1.57%)	127 (1.61%)	18 (0.72%)	23 (0.92%)
Asian	156 (0.89%)	155 (0.88%)	10 (0.85%)	10 (0.85%)	92 (1.16%)	90 (1.14%)	25 (1.00%)	24 (0.96%)
Native Hawaiian or Other Pacific Islander	21 (0.12%)	20 (0.11%)	10 (0.85%)	10 (0.85%)	10 (0.13%)	10 (0.13%)	10 (0.40%)	10 (0.40%)
American Indian or Alaska Native	38 (0.22%)	30 (0.17%)	10 (0.85%)	0 (0.00%)	21 (0.27%)	14 (0.18%)	10 (0.40%)	10 (0.40%)
Vitiligo	668 (3.80%)	27 (0.15%)	31	≤ 10**	381 (4.82%)	12 (0.15%)	66 (2.65%)	0 (0.00%)

Among patients receiving PD-1 inhibitor therapy (n=17,563 per cohort), those with MM had a significantly higher risk of developing vitiligo than matched non-MM patients (RR: 24.841; 95% CI: 16.917-36.447; p<0.0001). A similar pattern was observed among patients treated with IFN-α2b (n=1,182 per cohort), in whom MM was associated with an increased risk of vitiligo (RR: 3.103; 95% CI: 1.528-6.299, p=0.0009). Vitiligo occurred in 2.65% of patients receiving BRAF/MEK inhibitors with MM (n=2,491 per cohort), whereas no cases were identified among matched patients without MM; therefore, a relative risk could not be calculated for this comparison. In patients treated with CTLA-4 inhibitors (n=7,910 per cohort), MM was again associated with a significantly elevated risk of vitiligo (RR: 32.042; 95% CI: 18.052-56.875, p<0.0001) (Table 2). 

**Table 2:** Incident Vitiligo Risk by Treatment Cohort. MM: malignant melanoma; RR: risk ratio; CI: confidence interval; PD-1i: programmed cell death protein 1 inhibitor; IFN‑α2b: interferon alpha-2b; CTLA-4i: cytotoxic T-lymphocyte-associated protein 4 inhibitor; BRAF/MEKi: B-Raf serine/threonine kinases and mitogen-activated protein kinase kinases inhibitors. †After propensity score matching; *Statistically significant values between cases and controls, defined at p<0.05. **As per TriNetX user guidelines, data with fewer than 10 patients is suppressed from view. Note: RR, 95% CI, and p-value were not calculated or reported for BRAF/MEKi because the matched non-melanoma control group had zero incident vitiligo events.

Treatment	MM Cases Vitiligo	MM Controls Vitiligo	RR	95% CI †	P-value †
PD-1i	668 (3.80%)	27 (0.15%)	24.84	16.92-36.48	<0.0001*
IFN‑α2b	31	≤ 10**	3.10	1.53-6.30	0.0009*
CTLA-4i	381 (4.82%)	12 (0.15%)	32.04	18.05-56.88	< 0.0001*
BRAF/MEKi	66 (2.65%)	0 (0.00%)	-	-	-

**Figure 1:**
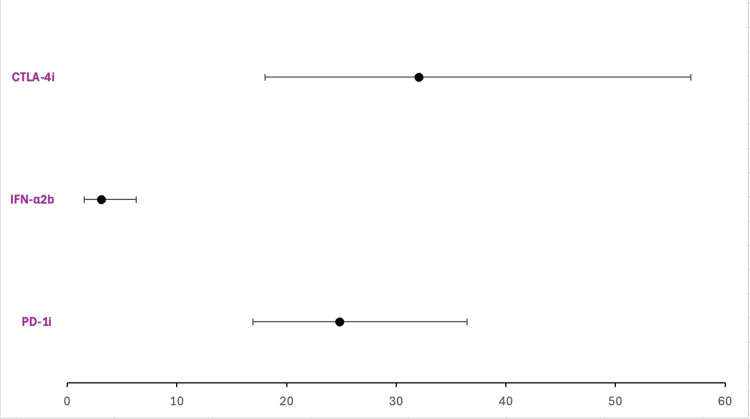
Forest Plot of Incident Vitiligo Risk Ratios by Treatment Cohort. CTLA-4i: cytotoxic T-lymphocyte-associated protein 4 inhibitor; IFN‑α2b: interferon alpha-2b; PD-1i: programmed cell death protein 1 inhibitor. Risk ratios (RR) are shown for cohorts in which RR and 95% confidence interval (CI) were calculable. BRAF/MEK inhibitors were not plotted because no incident vitiligo events occurred in the matched non-melanoma control group, preventing RR and CI estimation.

Figure 1 summarizes the direction and precision of the treatment-specific associations between malignant melanoma and incident vitiligo. Although the magnitude of association varied by therapy class, all confidence intervals remained entirely above the null value of 1.0, supporting a positive association across immune-modulating treatments. The wider confidence interval for CTLA-4 inhibitors reflects less precise estimation compared with the PD-1 inhibitor cohort, while the lower but still significant estimate for IFN-α2b suggests a more modest association. 

## Discussion

Across treatment cohorts, patients with melanoma demonstrated a higher incidence of vitiligo than matched patients without melanoma. These findings are compatible with immune-mediated mechanisms linking melanoma and vitiligo [4]. To draw inferences from these findings, we must first understand in detail the mechanism of action of each medication, which could be indicated for patients with or without MM. PD-1 inhibitors and IFN-α2b mainly exert systemic immunotherapy effects, whereas BRAF/MEK inhibitors exert their effect through targeted inhibition of tumor-specific replication pathways [5-7]. PD-1 inhibitors, such as pembrolizumab, function as immune checkpoint inhibitors, enhancing systemic T-cell-mediated antitumor responses [10]. When unaugmented, the immune system implements checkpoints that act to inhibit T-cell responses to tumors [11]. Within these checkpoint regions, PD-1 receptors are rapidly induced on naive T-cells following T-cell receptor engagement. Physiologically, this allows for PD-1 to counter T-cell activation in the presence of antigens, thereby protecting tissue from immune-mediated injury [12]. However, in states of repetitive T-cell stimulation (such as seen in cancerous states), PD-1 is expressed at high levels, leading to a dysfunctional state that ultimately diminishes T-cell response to tumors [11]. Precisely how PD-1 inhibitors exert their function is complex. However, it is sufficient to say they prevent PD-1 binding to PD-1 Ligand 1 and 2, allowing for restoration of cytotoxic CD8 T-cell response towards tumoral cells [13].

IFN-α2b is a widely biologically active cytokine that exhibits antiproliferative and antiviral effects by acting on the Janus-activated kinase/signal transducer activation of transcription (JAK-STAT) pathway. This pathway has broad effects, including proteins involved in the process of cell proliferation inhibition and cell apoptosis [6]. Induction of apoptosis in cells via the JAK-STAT pathway and subsequent tumor proliferation inhibition occurs in two main ways: Receptor signal transduction through tumor necrosis factor alpha and the release of cytochrome c via mitochondria [6,14]. Studies have also found that IFN-α2b therapy inhibits the activity of the transforming growth factor β (TGFβ)/Smad signaling pathway, thereby acting on the proliferation and migration of fibroblasts [15].

BRAF/MEK inhibitors function by blocking the mitogen-activated protein kinase (MAPK) pathway, which regulates melanocyte proliferation and survival [16]. In MM, activating BRAF mutations drive constitutive MAPK signaling, promoting tumor growth. Combination therapy is preferred to achieve more complete MAPK suppression, reduce melanoma progression, and limit resistance associated with single-agent treatment [17-18]. Beyond tumor control, BRAF/MEK inhibition can alter the tumor microenvironment by increasing melanoma antigen expression and enhancing CD8⁺ T-cell infiltration [19]. By reducing MAPK-driven survival signals, these inhibitors may sensitize both tumor and normal melanocytes to immune-mediated apoptosis [16-18].

CTLA-4 inhibitors are immune checkpoint blockers that enhance T-cell-mediated antitumor immunity by targeting CTLA-4, a receptor that normally downregulates early T-cell activation [19]. Inhibition of CTLA-4 allows for sustained T-cell receptor signaling, increased clonal expansion of effector T cells, and enhanced cytotoxic activity against tumor cells. CTLA-4 blockade also modulates regulatory T-cells, further reducing immune suppression [8,19]. By enhancing systemic T-cell activation and decreasing immune tolerance, CTLA-4 inhibitors may facilitate recognition of normal melanocytes [8].

The observed increased risk of vitiligo among melanoma patients receiving immune-based therapies such as PD-1 inhibition, IFN-α2b, and CTLA-4 inhibition underscores the complex interplay between melanoma pathophysiology and autoimmunity (Table 1). While BRAF/MEK inhibitor-related vitiligo cases were too limited to analyze, it was still statistically significant. The striking association with PD-1 inhibitors (RR: 24.84; 95% CI: 16.92-36.48), IFN-α2b (RR: 3.10; 95% CI: 1.53-6.30), and CTLA-4 inhibitors (RR: 32.042; 95% CI: 18.052-56.875) were all highly significant (p<0.0009), highlighting a consistent biological signal. These findings suggest that vitiligo is not merely a coincidental adverse event but may serve as a visible marker of immune activation against melanocytic antigens [20]. Importantly, this strengthens the concept that vitiligo in melanoma patients reflects an on-target immune response, linking clinical dermatologic manifestations to therapeutic efficacy and providing critical insight into the shared immunopathogenic mechanisms of melanoma and autoimmunity [20-21].

Our findings have limitations, including reliance on ICD-10 billing codes, which may not accurately capture all associations or be clinically validated [22]. The dataset could not distinguish classic vitiligo from vitiligo-like depigmentation, nor could it differentiate incident cases from preexisting disease. Another limitation is the inability to stratify data by length of treatment, prior or concomitant treatments, and time to presentation of vitiligo, as the reported data only showed cases after respective treatment for their MM, and reliance on patients to report symptoms of vitiligo [22-23]. Additionally, propensity score matching did not account for potentially important clinical confounders such as melanoma stage, prior autoimmune disease, dermatology follow-up, or healthcare utilization, which may have resulted in residual confounding. A prospective cohort study with clinical confirmation and detailed onset characterization, accounting for confounding variables such as prior treatments and environmental factors, would strengthen findings.

## Conclusions

These findings provide compelling information that may be useful in determining the link between MM and vitiligo by demonstrating the association between vitiligo development in MM versus non-MM patients receiving immune-activating therapies, especially PD-1 inhibitors, CTLA-4 inhibitors, and IFN-α2b. By comparing matched non-MM cohorts, we demonstrate an association between melanoma and subsequent vitiligo among patients receiving the same therapies. These findings may reflect shared immune mechanisms and warrant further investigation. Even among BRAF/MEK-treated patients, rare cases suggest that targeted therapy can indirectly prime immune recognition of melanocytes. These findings are consistent with a melanoma-associated melanocytic immune phenomenon, but they should be interpreted as hypothesis-generating rather than causal or biomarker validating. Prospective studies incorporating clinical assessment of vitiligo and immune profiling will be important to further define its relationship to treatment response.
